# Are Pensions and Disability Benefits concordant with Means and Health? A Tobit Approach to SHARE data from 50-to-89-year-olds covering 12 European Countries from 2015 to 2022

**DOI:** 10.21203/rs.3.rs-10795030/v1

**Published:** 2026-08-25

**Authors:** Hans Gevers

**Affiliations:** Estonian Business School

**Keywords:** public pension, disability pension, disability benefits, old-age pension, SHARE, means-testing, health, Tobit

## Abstract

Public pensions and benefits are a cornerstone for safeguarding citizens’ standard of living. This study focuses on the determinants of old-age pensions as well as disability pensions and benefits. The data is provided by the Survey of Health, Ageing and Retirement in Europe (SHARE), and covers people aged 50 to 89 years old for the period 2015 to 2022 in 12 European countries. The relationship between the determinants and old-age as well as disability pensions is estimated using Multi-effects and Random-Effects Tobit estimators with a minimum of 106,240 observations of minimum 47,832 respondents. The results indicate that both old-age and disability pensions appear to be limited complementary across countries. Old-age pensions are lower at lower self-reported health levels, while disability pensions and benefits appear to be higher. Moreover, the public payment schemes appear to account for the remaining available income of the beneficiary, implying that a given fairness is incorporated in the payment policies. Remarkably, both payments reveal the presence of a pension gender gap, potentially arising from a preceding wage gap. Overall, the study illustrates on a high level the fair allocation of available means to old-age as well as disability pensions across a substantial part of Europe.

## Introduction

The allocation of public pensions and disability benefits is a delicate balancing act aiming at meeting a considerable part of citizens’ needs. In particular for the older people, it implies a policy choice of distributing limited financial resources among the younger and older generations, working and retired citizens, or just as well among the able and less able people in society. This study documents the relationship between a series of individual features and public old-age pensions as well as disability pensions and benefits. Primarily, the influence of health and remaining income is of interest. The aim of the study is to document whether the pensions and benefits are distributed in a justifiable way. As such, the research questions to what extent the allocation of old-age pensions as well as disability pensions and benefits account for the beneficiary’s health and financial status. The purpose of the study is to contribute to the research field of Applied Health Economics by providing insight into the policy impact of payment schemes for older people across the European continent and over almost a decade. To enable reaching these targets, a sample is constructed based on SHARE data from Waves 6 to 9, i.e. 2015 to 2022, covering 12 European countries and citizens aged 50 to 89 years old. As a vast part of the respondents receive no pension or disability benefit, censoring is required, and subsequently, the Tobit estimation approach is most suitable. To theoretically support documenting public pensions and benefits, the work of John Rawls (1999) is referred to. First, for financial means in general, the expectation is that they are distributed fairly, and that a sacrifice for a few implies a greater benefit for many others. The Theory of Justice overarches the approach used in this paper as, according to Rawls, it regards “the way in which the major social institutions distribute fundamental rights and duties and determine the division of advantages from social cooperation”. In other words, the transfer of available public means, i.e. collected taxes and social contributions, from the entire population to specific groups like retirees or disabled. The latter disabled were explicitly excluded by Rawls from his social contract theory through capacity contracts. Nowadays political theory broadly covers disability with a variety of definitions and criteria. However, in practice the inclusion of people with disabilities is likely more nuanced. It regards adapting the material world to the disability, or modifying the disability itself. It regards considering viewpoints and attitudes reflecting the destructiveness or betterment of a disability. The social model of disability suggests that a disability is merely created by its surrounding, not a bodily difference. For example, a wheelchair user is at a given time merely disabled if a building only has stairs and no ramps (Arneil & Hirschmann, 2016, pp. 20,79, 99, 263). Similarly, disability benefits target overcoming the barriers or differences people with physical, mental, or cognitive limitations experience while participating in daily activities, in daily life. Overall, this outlined theoretical basis supports that public general and disability payments ought to be just, i.e. to a given extent according to available financial means and health status. Acknowledging this theoretical expectation, the subsequent paragraphs further explore the existing literature, specify the data used, define the methodology, and report the analysis outcome, discussion, as well as conclusion.

## Literature Review

The existing literature documents a variety of studies that illustrate the fairness of means redistribution within societies. A first important note is that the life quality insurance of older people can, to a certain extent, be considered as an economic load for the younger generation, i.e. less means reserved for families and children. Focacci (2023) notices this “predator-prey relationship” being driven by the governmental prioritisation of social support for the ageing population. She concludes that across a decade, the Austrian and Swiss governments favoured the older population, while Germany maintained a status quo. Similarly, del Carmen and Sosvilla-Rivero (2025) conclude that higher dependency and subsequently higher poverty rates occur when the proportion of the population being 65 or older is larger relative to the working population. Furthermore, they highlight that also the macro-economic situation is most relevant for poverty. Despite these constraining relationships, governments are expected to redistribute, e.g., through public pensions, with the aim to overcome inequality. The rising importance of private pensions ought to be recognised as a crucial disruptor of this valuable aim as it tends to reinforce inequality (Doctrinal, 2024). Besides overarching influences, social systems inherently and historically differ. Nádasi and Kovács (2026) note how European pension systems vary, ranging from the Northern active aging model to the Southern transfer model. Some of these systems face an additional financial burden of early retirement, while others conceal a more explicit gender pension gap. Nádasi et al. conclude that there is “no single solution to the European pension crisis”. In turn, this conclusion suggests that, within Europe, retirees live at varying life standards. Jajko-Siwek (2024) investigates retirees in Poland, Spain and Denmark based on SHARE data. With Machine Learning techniques, they document 167 features that determine the retirees’ ability to make ends meet. Evidently, financial means are the dominant determinant, with an explicit difference between the three countries. In Denmark, with its reputable welfare system, retirees appear to manage best, while in Poland, a relatively larger part of retirees have difficulties making ends meet. Additionally, for Danish, housing costs are a primary concern, while for Spanish and Polish retirees, food expenditure is the main concern. These findings steer towards prioritising fairness on a national level, i.e. a fair distribution of pensions according to available means as part of sustaining an efficient national pension system. Padlowska (2025) evaluates whether the pension contributions level off with the expenditures in Poland and Germany. Neither appears sustainable based on national data from 2021 to 2024. The sustainability of the national social systems also impacts the differing national healthcare systems. As such, pension payments are found not only to affect health, they also directly impact the healthcare system itself. An et al. (2016) find an increase in public pension to trigger an increase in health expenditure of up to 30% across 21 countries around the world. In general, this mechanism obeys the tweaks of the direct relationship between financial status and health. Bialowolski et al. (2025) relate worse health to worse financial status, with debts, depression, functional and mobility limitation as an additional health burden. Moreover, not merely the value of the financial means appears important, also their subjective or perceived value is relevant. These findings rely on SHARE data from 2004 to 2022. Aligning with health, various other behavioural aspects are considered relevant for pensions. For example, the savings as well as real estate of retirees are known to determine life quality. The retirees’ willingness to decumulate wealth is expected to depend on the intent to leave bequest, as illustrated with SHARE data of 11 European countries by Yuji Horioka and Ventura (2024). Additionally, the mere take-up of means shapes social payment policies. Bütler et al. (2017) illustrate how individual Swiss pension reserves are consumed as a lump sum, and when depleted at a certain level, they are replaced by means-tested benefits. To conclude, demographic and personal characteristics are a second important influence on means redistribution.

On top of the two dominant influences, pension payments to older people are in particular dependent on the occurrence of physical, mental, or cognitive limitations, i.e. disabilities. Morris (2021) documents for nine countries with 2015-SHARE data that people dependent on disability benefits are twice as likely to remain in need. He finds this need more explicit in Austria, Spain, Italy, France, and Belgium compared to Denmark, Sweden, Germany, and Switzerland. The same data reveals that a significant part of the sample with work-disabilities receives no disability benefits. Furthermore, constraining the eligibility criteria of disability benefits is expected to impact health. Jensen et al. (2018) rely on SHARE data to conclude that policy reforms in Denmark and Sweden increased stress for people with moderate to severe health problems due to receiving reduced or no means-tested benefits. Lastly, other influences may occur. For example, remaining single implies missing out on the economic benefits of marriage or cohabitation. Across a few decades, Namatovu et al. (2020) find that Swedish recipients of disability pensions have a lower tendency to form partnerships, potentially due to a desire to be less financially dependent. In conclusion, a variety of subinfluences impacting the needs of disabled confirm how financial redistribution is a challenge to fairness in society.

### Data

The longitudinal analysis in this study relies on data from SHARE (Bergmann et al., 2019; Börsch-Supan et al., 2013). The latter is a recurring health survey that inquires European citizens about the effects of health, social, economic and environmental policies. The data used for this study covers Waves 6 to 9, and 12 countries in Europe, being Austria, Germany, Sweden, Spain, Italy, France, Denmark, Greece, Switzerland, Belgium, Czechia, and Poland (SHARE-ERIC, 2024a, 2024b, 2024c, 2024d). The dataset feeds the regressions with a minimum of 106,240 observations of minimum 47,832 respondents. Table 1 describes the variables in the dataset.

The variable Wave year refers to 2015, 2017, 2020, or 2022, during which a maximum of 47,945 respondents of 12 European countries (with the variable Country referring to SHARE country numbers) report a set of maximum ten personal features. First, Age, which ranges from 50 to 89, with the average respondent being 70 years old. Second, the Years of education a respondent received range from zero to 30, with an average of 11 years. Third, Gender notes male (1) or female (2). Fourth, the health status is documented by Self-perceived health, which ranges from excellent (1) to poor (5). Fifth, the Current job situation consists of five categories, i.e. retired, unemployed, (self-)employed, permanently sick, and homemaker. Sixth, the variable Housing with five categories specifies if the respondent is rent-free, an owner, a tenant, a subtenant, or a tenant with a cooperative. Seventh, the relational status of the respondent is covered by being not single or single. Eighth, Old-age pensions are old-age, early retirement, and survivor pensions which range from zero to 252,000 euros. The average amounts 9,122 euros. Nineth, Disability pensions are disability pensions or benefits. They amount 314 euros on average with a maximum of 224,740 euros. Tenth, the total household income, which also includes one or both previous amounts, on top of other forms of income like private occupational pensions, unemployment benefits, sickness benefits, income from other household members, and the like. This total income amounts 31,307 euros on average, and notes a maximum of 252,700 euros. To counter the extremes, the respondents with an original total income larger than four times the standard deviation from the mean income are excluded from the sample. A variable Income quartile is constructed by deducting the Old-age pensions or Disability pensions from the Total household income. It covers four categories, from Lowest to Highest, which reflect the income the respondent is expected to have available if needed, besides the Old-age pension or Disability pension. It provides a benchmark to verify if the pension payments account for the remaining means the respondent has available.

From the list of variables, two are used as independent variables, namely Old-age pensions as well as Disability pensions and benefits. Both variables are used denominated in euros. Extreme values are trimmed four standard deviations above the mean to avoid estimation difficulties or inaccuracies for the Tobit estimators. Yet, a given inaccuracy is a priori expected given that SHARE data predominantly relies on self-reported input. Schimmel, Hyde, and Harrati (2023) illustrate how self-reported sizes of disability and supplemental benefits do not exactly align with officially recorded ones across the United States. As the exact amounts are not interpreted in this study, this inaccuracy is expected not to change the conclusions drawn from the estimates. Additionally, to illustrate sample quality, the independent variables record a sufficient number of observations in the category of interest, namely the pension or benefit. Table 2 illustrates the number of zero and non-zero values for both Old-age pensions and Disability pensions (and benefits).

Table 2 reveals the relatively lower number of reported disability pensions or benefits. This is evident as it regards a minority group in the total sample SHARE periodically surveys. Lastly, Table 3, on the next page, tabulates the Spearman’s Rank correlation coefficients. Three coefficients are worth noting. Age as well as Current job situation correlate strongly with Old-age pension. Given that the latter is an independent variable, the high correlations are not an issue. Additionally, Age and Current job situation correlate, yet this correlation remains below a 0.600 threshold.

To conclude, the selected set of variables is expected to enable revealing to what extent the amount of old-age and disability pensions accounts for health and financial means while controlling for other individual characteristics.

## Methodology

The data is modelled in Stata 19.5 Basic Edition with Multi-effects and Random-Effects Tobit estimators, which both can handle censored data. Both estimators allow to identify each respondent on the panel level as each respondent is carried forward across the time horizon. Random effects are preferred as SHARE follows a random sampling process for its survey. The time dimension is integrated in the estimation as a categorical variable to enable the estimation. The Multi-effects Tobit estimator is used as it allows using sampling weights. The latter are calibrated longitudinal weights which were estimated according to the SHARE procedure for the specific time horizon and with updated demographic data from Eurostat (De Luca & Rossetti, 2018; 2026a, 2026b). The Random-Effects Tobit estimator regresses the natural logarithm of the dependent variables (while keeping the zero values) to enable convergence. The interpretation of the outcome of the estimators is a priori limited to their sign, as it suffices to evaluate the impact of each characteristic on the old-age or disability pension.

## Results

The estimation results of the Multi-effects and Random-Effects Tobit estimators are revealed in Table 4 on the next page. The Random-Effects Tobit estimators report a non-zero panel-level variance component as well as a likelihood-ratio test significantly different from zero. Both indicate that the panel estimators are preferred to the pooled, i.e. standard Tobit estimator.

The results in Table 4 aim to explain the variation in the Disability pensions/benefits and Old-age, early retirement, and survivor pensions, subsequently referred to as, respectively, Disability pensions and Old-age Pensions. Ten individual characteristics are tabulated. Columns two and four cover the Multi-effects Tobit estimators and rely on the dependent variables denominated in euros, while columns three and five cover the Random-Effects Tobit estimators with the dependent variables the natural logarithm of the dependent variables denominated in euros. The latter is required to enable the estimators to converge. The effect of the characteristics is discussed in the following paragraph.

First, respondents being single report a higher old-age pension on average, while disability pensions are lower for singles. It is sound that the former lack the economic benefits from marriage or cohabitation, while the latter suggests that singles may have more difficulty living independently, yet continue living independently when in a relationship. This is not a priori discrimination against non-independent singles, as they are most likely living in a nursing home. In the data filtered for this study, none of the respondents resides in a nursing home, implying that their disability benefits are not reflected in this estimate. Second, regarding housing, the respondents who are homeowners appear to receive higher pensions overall. A plausible explanation is that home ownership relates to a full working career and subsequently full public pension rights. On top of the 75 per cent of the sample being a homeowner, the remaining appear to live at home, or rent from a cooperative or landlord. A small group of 38 respondents report being subtenants, and they receive a slightly higher disability pension than the homeowners. For this specific group, the surplus potentially, given the effect is not significant, supports renting accommodation suitable for disabled, which is not categorised as a nursing home. Third, females appear to receive less old-age pension as well as disability pension. For the former, a potential wage gap may spill over into the pension payment schemes through preceding reserved pension rights. For the latter, a sound explanation is not readily available, given that the used dataset is rather gender balanced on several of the available health indicators. The occurrence of an income gap is expectable as it is well documented, e.g., by Helmdag and Väänänen (2025). Fourth, the work status reports a similar trend across both old-age and disability pensions. Solely unemployed report a lower pension than the employed or self-employed, which suggests that they receive a survivor instead of full pension due to fewer working years. Retirees receive the highest old-age pension, while the permanently sick report the highest disability pension. Note that of the 19 per cent employed or self-employed respondents in the sample, only 18 respondents report a non-zero pension. Fifth, aging seems, on the one hand, to increase old-age pensions. This most likely reflects the adaptation of pensions to the cost of living. On the other hand, ageing decreases the disability pensions, which results most likely from updating public payment schemes. Sixth, an increase in years of education provides a higher old-age pension. Soundly, higher education relates to higher wages and subsequently more accumulated pension rights. The same increase negatively affects the disability pension. A possible explanation may be that higher educated less likely fill in blue-collar positions and may be less exposed to risky or heavy-duty tasks. Seventh, one of two variables of interest, i.e. self-perceived health status, is reported. Both old-age and disability pensions appear to be limited complementary across countries. The old-age pension gradually decreases when health deteriorates, yet the disability pension or benefit increases according to health status. The spillover between both pensions is limited as, of the respondents reporting a poor health status, roughly six per cent receive old-age pensions, while 0.77 per cent receive disability benefits and 0.18 per cent receive both. A sound explanation for the lower old-age pensions of those with lower self-reported health levels is that the preceding working years are also limited due to health limitations, which subsequently bound the public pension rights.

Eighth, the time dimension is reflected by including a categorical covering each regressed wave of SHARE. For the old-age pension, the effects are not significant. For the disability pension, the effects reflect a modest upward trend. Potentially, the time variable, instead of the Age variable, captures the adaptation of the pension to the cost of living. Nineth, the country variable captures the difference in pension payments between the 12 countries. For the old-age and disability pensions, the Southern and Eastern European countries (being Czechia, Poland, Greece, Spain, and Italy) report lower pension payments. The remaining, rather Western European countries, appear to have more generous schemes. This contrasting difference can primarily be explained by substantial differences in the cost of living, as well as by the difference in care provision. In particular, Eastern regions are expected to be exposed to higher informal care needs (Cattaneo et al., 2025). Lastly, the second of the two prior variables of interest, i.e. the remaining income, is discussed. The old-age and disability public pension payment schemes reflect a given fairness as both decrease when the remaining available income increases. For the old-age pensions, the difference between the lower half and upper half cohort of respondents is more explicit, suggesting that the former sets the standard for the pensions and the latter receives relatively less, given that the individual remaining income, e.g. obtained through private pensions, is taken into account for setting the public pension. For the disability benefits, the lowest group is explicitly favoured. This similarly confirms safeguarding a standard of living for those in need. To conclude, the ten variables explicitly and soundly document the most important determinants of old-age and disability pensions.

On top of the interpretation of the coefficients, a selection of margin plots is documented to visualise the dependence of pensions and benefits on means and health.

**Figure 1:**
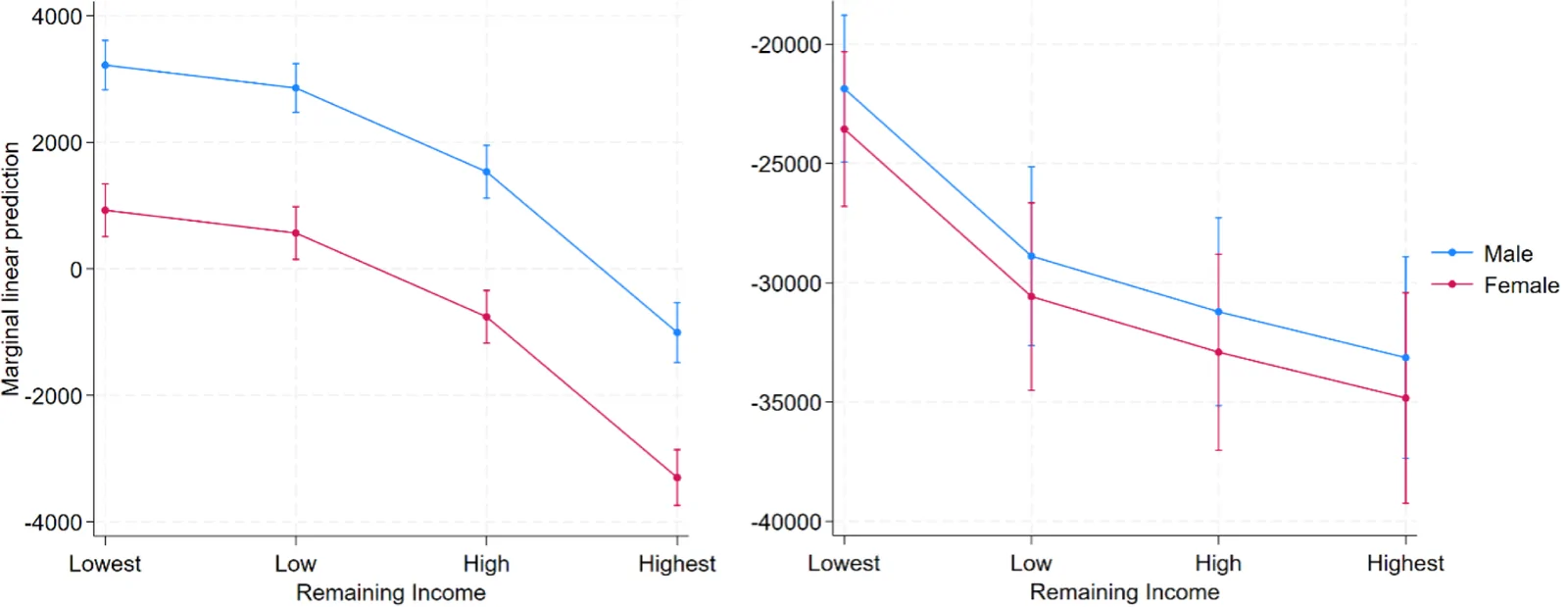
Margins relative to Gender, and Remaining Income ☒ Old-age Pensions (left) and Disability Pensions (right)

Figure 1 illustrates that both Old-age pensions, plotted on the left-hand side, as well as Disability pensions, plotted on the right-hand side, are relatively lower for those with more means available. Both graphs explicitly confirm the pension gap in favour of males, potentially arising from a preceding wage gap.

**Figure 2:**
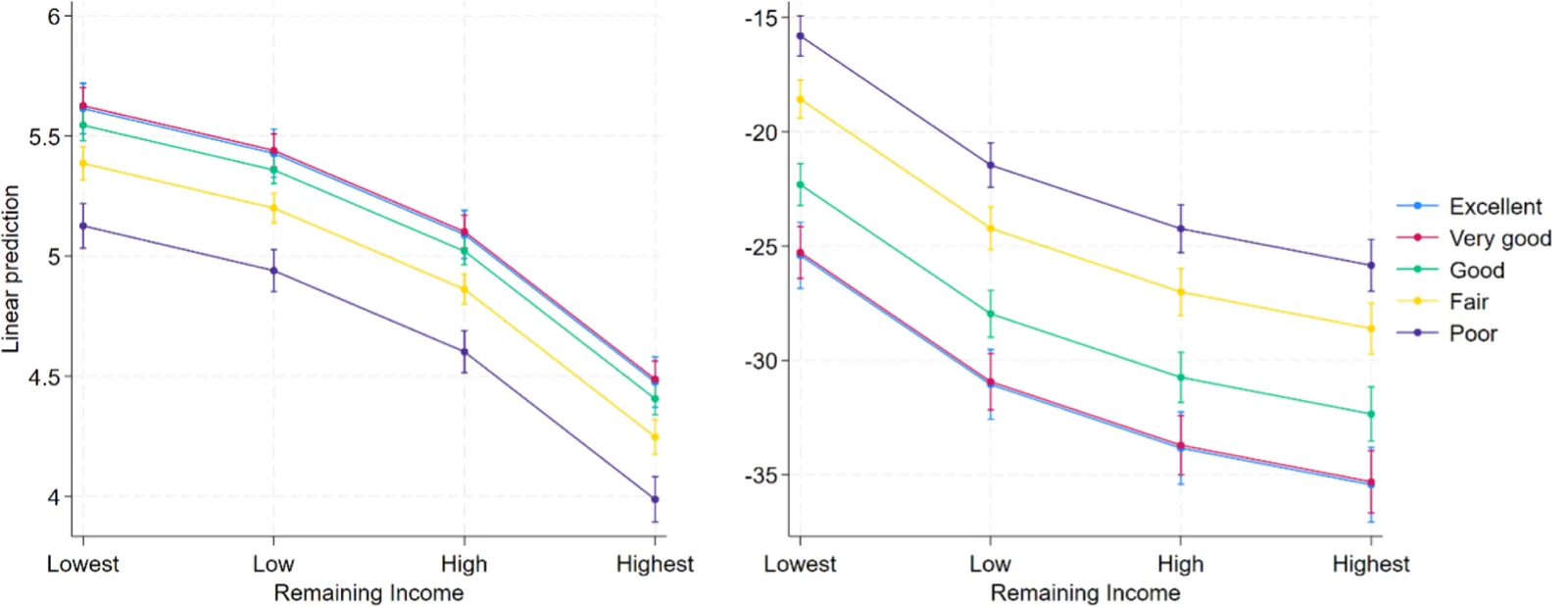
Margins relative to Health, and Remaining Income ☒ Old-age Pensions (left) and Disability Pensions (right)

Figure 2 reveals, plotted on the left-hand side, that lower health levels relate to lower old-age pensions. For the disability pensions, plotted on the right-hand side, the inverse is true, i.e. higher amounts relate to lower health levels. A spillover effect is limited. Merely 0.18 per cent of the respondents with poor self-perceived health status receive a disability benefit on top of their old-age pension. Note that the scale of the Y-axis for both Figs. 1 and 2 is less informative, given that the right-hand side relies on the natural logarithm of the dependent variable Disability pension, while the left-hand side relies on the raw monetary amount. Overall, the marginal predictions visualise how pension allocation accounts for the respondent’s available means as well as health status. However, a clear gender gap is identified.

## Discussion

This study documents the influence of ten individual characteristics on the amount of old-age pensions as well as disability pensions and benefits. Although the documented insights support the fair allocation of the available public resources on a high level, they are bound by some notable constraints. First, a high-quality survey like SHARE is expected to remain exposed to interview effects, which have not been accounted for in this study (Beullens & Loosveldt, 2016). Second, the study supports a European divide as found in healthcare (Manolova et al., 2019). Yet, it neglects the same divide in tax policies (Cieślukowski, 2024). As such, the differences between the countries are potentially less explicit. Third, the amount of old-age pensions as well as disability pensions and benefits is benchmarked against the categorised remaining income. The latter neglects the value of savings or other property. Potentially, this introduces bias into the analysis for respondents who report a relatively low income yet have accumulated a considerable amount of wealth through saving or homeownership. In conclusion, the limitations support interpreting merely the signs of the regression estimates in order to conclude on the impact of payment scheme policymaking across countries.

## Conclusion

This study provides insight into the determinants of old-age pension and disability pensions and benefits based on SHARE data from citizens from 12 European countries aged 50 to 89 years old. It documents the impact of ten identified individual characteristics occurring across almost a decade of payments. Notably, higher amounts are allocated to singles and to males. The former may back independent living, while the latter likely arises from a preceding gender wage gap. Moreover, the study supports a European divide where the Northern and Western countries appear to have more generous payment schemes than the Southern and Eastern ones. This divide likely originates in a difference in cost of living. Lastly, an answer to the research question is illustrated. As such, ceteris paribus, on average, and across countries, old-age pensions as well as disability pensions and benefits tend to account for health and financial status.

## Figures and Tables

**Table 1:** Descriptives of variables

Variable	Unique	Mean	Minimum	Maximum
Wave year	4	-	2015	2022
Country	12	-	11	29
Age	40	70	50	89
Years of education	30	11	0	30
Gender	2	-	1	2
Self-perceived health	5	-	1	5
Current job situation	5	-	1	5
Housing	5	-	1	5
Single	2	-	0	1
Old-age pensions (in euros)	19,016	9,122	0	252,000
Disability pensions (in euros)	1,708	314	0	224,740
Total household income (in euros)	53,847	31,307	0	252,700

Old-age pensions = Old-age, early retirement, and survivor pensions; Disability pensions = Disability pensions/benefits

Number of observations: 106,276. All continuous variables are rounded for tabulation.

**Table 2:** Zero and non-zero pensions per country

Country	Old-age Pensions		Disability Pensions	
	Zero	Non-zero	Zero	Non-zero
Austria	1,312	5,244	6,320	236
Germany	3,318	6,675	9,647	346
Sweden	2,358	6,340	8,409	289
Spain	3,740	5,481	8,926	295
Italy	4,738	6,360	10,675	423
France	1,954	6,569	8,271	252
Denmark	3,284	5,165	8,086	363
Greece	5,419	6,350	11,532	237
Switzerland	1,686	4,501	6,047	140
Belgium	3,978	7,360	10,822	516
Czechia	1,608	8,449	9,671	386
Poland	1,031	3,356	4,124	263

Note: Old-age Pensions are Old-age, early retirement, and survivor pensions; Disability Pensions are Disability pensions/benefits

**Table 3:** Spearman's Rank Correlation Coefficients

1	Variables	1	2	3	4	5	6	7	8	9	10	11	12
	Wave year	1.000	-	-	-	-	-	-	-	-	-	-	-
2	Country	0.010	1.000	-	-	-	-	-	-	-	-	-	-
3	Housing	0.003	−0.065	1.000	-	-	-	-	-	-	-	-	-
4	Single	0.046	0.030	0.224	1.000	-	-	-	-	-	-	-	-
5	Old-age pensions	0.159	−0.098	0.050	0.136	1.000	-	-	-	-	-	-	-
6	Disability pensions	−0.026	0.011	0.037	0.019	−0.152	1.000	-	-	-	-	-	-
7	Gender	0.012	0.017	0.050	0.194	−0.151	−0.014	1.000	-	-	-	-	-
8	Age	0.215	−0.047	0.085	0.193	0.519	−0.115	−0.034	1.000	-	-	-	-
9	Years of education	0.043	0.059	−0.043	−0.060	0.000	−0.029	−0.068	−0.221	1.000	-	-	-
10	Self-perceived health	0.012	0.002	0.086	0.100	0.042	0.136	0.022	0.250	−0.217	1.000	-	-
11	Current job situation	−0.126	−0.023	−0.060	−0.066	−0.656	0.108	0.173	−0.499	−0.007	−0.079	1.000	-
12	Total household income	0.035	−0.230	−0.068	−0.322	0.267	−0.020	−0.102	−0.149	0.272	−0.244	−0.027	1.000

Old-age pensions = Old-age, early retirement, and survivor pensions; Disability pensions = Disability pensions/benefits

**Table 4:** Multi-effects and Random-Effects Tobit estimators

VARIABLES	Old-age Pensions		Disability Pensions	
	MeTobit	XtTobit	MeTobit	XtTobit
Social: Not single (base)				
Single	1,677.159***	0.665***	−1,386.745*	−1.138***
	(207.538)	(0.035)	(574.024)	(0.298)
Housing: Owner (base)				
Cooperative	−676.540*	−0.301***	−531.846	−0.060
	(288.275)	(0.074)	(917.614)	(0.680)
Tenant	−1,431.749***	−0.327***	−725.777	0.564
	(208.587)	(0.038)	(620.895)	(0.330)
Subtenant	−1,019.121	−0.440***	93.176	1.332
	(650.203)	(0.134)	(1,667.222)	(1.120)
Rent free	−1,159.057***	−0.265***	−1,194.672	−1.097*
	(204.147)	(0.049)	(890.134)	(0.512)
Gender: Male (base)				
Female	−2,293.196***	−0.363***	−1,694.741***	−1.644***
	(155.560)	(0.029)	(467.005)	(0.266)
Work: (Self-)Employed (base)				
Retired	23,831.330***	9.490***	8,485.463***	6.396***
	(464.257)	(0.047)	(970.904)	(0.413)
Unemployed	−2,506.636*	−1.778***	−13.885	−0.920
	(1,188.912)	(0.159)	(1,810.706)	(0.877)
Permanently sick	4,797.903***	1.116***	25,345.672***	21.825***
	(675.913)	(0.097)	(1,569.987)	(0.525)
Homemaker	10,554.848***	3.059***	3,414.922**	2.931***
	(511.632)	(0.063)	(1,218.939)	(0.589)
Age	365.909***	0.141***	−517.747***	−0.526***
	(12.317)	(0.002)	(47.053)	(0.020)
Years of education	412.044***	0.025***	−170.052**	−0.218***
	(19.298)	(0.003)	(55.737)	(0.033)
Health: Excellent (base)				
Very good	314.808	0.012	−81.728	0.124
	(317.825)	(0.051)	(1,304.089)	(0.653)
Good	−60.710	−0.069	4,003.913**	3.095***
	(305.070)	(0.049)	(1,299.752)	(0.610)
Fair	−1,001.561**	−0.228***	7,599.210***	6.833***
	(312.723)	(0.052)	(1,376.529)	(0.619)
Poor	−1,782.527***	−0.488***	9,339.766***	9.598***
	(343.497)	(0.061)	(1,501.192)	(0.662)
Year: 2015 (base)				
2017	226.034	0.074*	1,404.666**	0.324
	(119.100)	(0.031)	(488.509)	(0.333)
2020	−173.611	−0.096***	1,952.638***	0.845**
	(133.607)	(0.025)	(515.252)	(0.262)
2022	231.626	−0.034	2,117.017***	0.616*
	(128.182)	(0.025)	(558.852)	(0.272)
Country: Sweden (base)				
Austria	5,523.920***	0.686***	−3,270.706**	−1.977**
	(328.454)	(0.073)	(1,077.934)	(0.659)
Germany	2,737.264***	0.528***	−6,521.866***	−5.547***
	(275.971)	(0.068)	(989.176)	(0.640)
Spain	−1,432.483***	−0.613***	−11,327.526***	−8.716***
	(389.370)	(0.071)	(1,346.479)	(0.675)
Italy	−411.079	−0.583***	−9,455.636***	−5.546***
	(280.524)	(0.069)	(1,103.046)	(0.617)
France	6,054.693***	0.852***	−8,210.534***	−5.075***
	(276.014)	(0.070)	(1,100.161)	(0.653)
Denmark	97.710	0.412***	−1,195.162	−1.799**
	(248.124)	(0.071)	(884.212)	(0.646)
Greece	−3,923.223***	−1.071***	−13,753.315***	−10.834***
	(284.935)	(0.071)	(1,291.401)	(0.698)
Switzerland	11,202.475***	1.789***	−866.977	−2.857***
	(359.285)	(0.076)	(1,319.107)	(0.774)
Belgium	9,686.116***	0.444***	−4,538.104***	−2.740***
	(483.987)	(0.066)	(1,011.974)	(0.577)
Czechia	−6,974.265***	−0.421***	−7,001.301***	−7.457***
	(334.852)	(0.069)	(1,386.911)	(0.633)
Poland	−6,311.033***	−0.590***	−14,754.066***	−10.769***
	(312.551)	(0.089)	(1,336.018)	(0.781)
Income quartile: Lowest (base)				
Low	−360.478	−0.186***	−7,021.274***	−5.652***
	(186.807)	(0.034)	(679.518)	(0.314)
High	−1,684.797***	−0.524***	−9,352.711***	−8.435***
	(223.309)	(0.040)	(846.349)	(0.388)
Highest	−4,225.773***	−1.139***	−11,276.352***	−10.036***
	(269.981)	(0.047)	(1,040.876)	(0.458)
Constant	−41,484.159***	−11.141***	12,120.722***	14.805***
	(1,007.905)	(0.164)	(2,867.681)	(1.489)
Observations	106,240	106,276	106,240	106,276
Number of respondents	47,832	47,862	47,832	47,862
Robust standard errors	Yes	No	Yes	No

*** p<0.001,

** p<0.01,

* p< 0.05

## Data Availability

To provide insight into the longitudinal analysis completed in this article, do- and log-files of Stata are available via https://github.com/hansgevers/thefairnessofpensions.
